# Malnutrition and related risk factors in older adults from different health-care settings: an *enable* study

**DOI:** 10.1017/S1368980019002271

**Published:** 2019-08-27

**Authors:** Eva Kiesswetter, Miriam G Colombo, Christa Meisinger, Annette Peters, Barbara Thorand, Rolf Holle, Karl-Heinz Ladwig, Holger Schulz, Eva Grill, Rebecca Diekmann, Eva Schrader, Peter Stehle, Cornel C Sieber, Dorothee Volkert

**Affiliations:** 1 Institute for Biomedicine of Aging, Friedrich-Alexander Universität Erlangen-Nürnberg, Kobergerstrasse 60, 90408 Nuremberg, Germany; 2 Institute of Epidemiology, Helmholtz Zentrum München, German Research Centre for Environmental Health, Neuherberg, Germany; 3 MONICA/KORA Myocardial Infarction Registry, Central Hospital of Augsburg, Augsburg, Germany; 4 Institute for Epidemiology, Ludwig-Maximilians-University Munich, UNIKA-T Augsburg, Augsburg, Germany; 5 Institute of Health Economics and Health Care Management, Helmholtz Zentrum München, German Research Centre for Environmental Health, Neuherberg, Germany; 6 Department of Psychosomatic Medicine and Psychotherapy, Klinikum rechts der Isar, Technische Universität München, Munich, Germany; 7 Institute for Medical Information Processing, Biometrics and Epidemiology (IBE), and German Center for Vertigo and Balance Disorders, Ludwig-Maximilians-University Munich, Munich, Germany; 8 Department of Health Services Research, Carl von Ossietzky Universität Oldenburg, Oldenburg, Germany; 9 IEL-Nutritional Physiology, Universität Bonn, Bonn, Germany

**Keywords:** Malnutrition, Risk factor, Older people, Setting

## Abstract

**Objective:**

The origin of malnutrition in older age is multifactorial and risk factors may vary according to health and living situation. The present study aimed to identify setting-specific risk profiles of malnutrition in older adults and to investigate the association of the number of individual risk factors with malnutrition.

**Design:**

Data of four cross-sectional studies were harmonized and uniformly analysed. Malnutrition was defined as BMI < 20 kg/m^2^ and/or weight loss of >3 kg in the previous 3–6 months. Associations between factors of six domains (demographics, health, mental function, physical function, dietary intake-related problems, dietary behaviour), the number of individual risk factors and malnutrition were analysed using logistic regression.

**Setting:**

Community (CD), geriatric day hospital (GDH), home care (HC), nursing home (NH).

**Participants:**

CD older adults (*n* 1073), GDH patients (*n* 180), HC receivers (*n* 335) and NH residents (*n* 197), all ≥65 years.

**Results:**

Malnutrition prevalence was lower in CD (11 %) than in the other settings (16–19 %). In the CD sample, poor appetite, difficulties with eating, respiratory and gastrointestinal diseases were associated with malnutrition; in GDH patients, poor appetite and respiratory diseases; in HC receivers, younger age, poor appetite and nausea; and in NH residents, older age and mobility limitations. In all settings the likelihood of malnutrition increased with the number of potential individual risk factors.

**Conclusions:**

The study indicates a varying relevance of certain risk factors of malnutrition in different settings. However, the relationship of the number of individual risk factors with malnutrition in all settings implies comprehensive approaches to identify persons at risk of malnutrition early.

Malnutrition is widespread in the older population and leads, if it is untreated, to numerous negative clinical consequences such as functional decline, in-hospital complications, increased mortality and reduced quality of life^(^ ^1^ ^–^ ^4^ ^)^. In a recently published meta-analysis including 240 studies with 113 967 older adults from different settings, prevalence of malnutrition according to the Mini Nutritional Assessment was described to be 3 % in community-dwelling adults, 6 % in older outpatients, 9 % in older adults receiving home care and 18 % in nursing home residents^(^ ^5^ ^)^. However, published prevalence data on malnutrition varies widely between studies, even within specific settings^(^ ^6^ ^–^ ^8^ ^)^, probably due to different sampling characteristics and diagnostic criteria being used.

Age-related physiological changes, limitations in physical and mental function, health and social aspects are discussed to be relevant individual factors in the aetiology of malnutrition in older people^(^ ^9^ ^–^ ^11^ ^)^. However, studies investigating individual risk factors of malnutrition showed inconsistent results^(^ ^12^ ^–^ ^14^ ^)^. These inconsistencies may be partly due to different definitions of malnutrition, diverse assessment methods and different sets of investigated risk factors. Furthermore, due to different health, functional and living conditions, risk profiles of malnutrition in older adults may also vary depending on the investigated setting. Studies systematically comparing malnutrition risk profiles of older adults from different settings are scarce. Meijers *et al*. compared hospital, home care and nursing home settings and focused primarily on the question of which disease-related factors are associated with malnutrition^(^ ^15^ ^)^. In the home care setting, cancer, not having diabetes and gastrointestinal diseases were identified as risk factors; in the nursing home setting, female gender and dementia; and in the hospital setting, infection, cancer, dementia, blood diseases, chronic obstructive pulmonary disease and gastrointestinal diseases. Shatenstein *et al*. compared community-dwelling older adults and institutionalized older people (not further specified) by investigating risk factors of malnutrition from different domains^(^ ^16^ ^)^. Loss of appetite as well as loss of interest in life were identified as risk factors of malnutrition in community-dwelling older people and loss in interest in life as well as frailty in institutionalized older people. Such approaches including older adults from different settings and covering multiple domains of potential risk factors could help to identify setting-specific differences in malnutrition risk profiles of older people and could contribute to the development of differentiated and targeted prevention and intervention concepts.

As the origin of malnutrition is multifactorial^(^ ^9^ ^,^ ^10^ ^)^, older people are usually affected by several risk factors of malnutrition. However, studies focus mainly on the identification of specific risk factors of malnutrition^(^ ^12^ ^–^ ^14^ ^)^ but do not consider that the simultaneous occurrence of several risk factors may aggravate the risk of malnutrition.

The primary aim of the present study, a secondary data analysis, was to identify setting-specific risk profiles of malnutrition in community-dwelling older adults, patients of a geriatric day hospital, receivers of home care and nursing home residents by applying uniform definitions of malnutrition and potential individual risk factors from the domains of demographics, health, mental and physical functioning, dietary intake-related problems and dietary behaviour. A secondary aim was to investigate the association between the number of individual risk factors and the presence of malnutrition in these four settings.

## Methods

### Study design and study samples

The current secondary data analysis was a sub-project within the *enable* research cluster (http://www.enable-cluster.de/) and focused on four German cross-sectional studies with older adults at least 65 years of age from four different settings, conducted by the Institute for Biomedicine of Aging (Nuremberg) or the Helmholtz Zentrum München. The studies were selected because of similar inclusion and exclusion criteria as well as identical or comparable assessment methods.

The first study was based on a sex- and age-stratified random sample of older community-dwelling adults (CD, *n* 1079) living in the region of Augsburg and having German citizenship (KORA-Age, Cooperative Health Research in the Region of Augsburg)^(^ ^17^ ^)^. The data used in the current secondary data analysis refer to the baseline examination in 2009.

The second study comprised patients of a geriatric day hospital (GDH, *n* 198) and was performed in Nuremberg in 2012^(^ ^18^ ^)^. Exclusion criteria were discharge within the first 2 d after admission to the GDH and inability to communicate due to severe cognitive impairment, psychological problems, or language or hearing problems.

The third study (ErnSiPP ‘Ernährungssituation von Seniorinnen und Senioren mit Pflegebedarf in Privathaushalten’) investigated older adults receiving home care (HC, *n* 353) in three German cities (Bonn, Nuremberg, Paderborn) in 2010^(^ ^19^ ^)^. Participants lived in a private household, were allocated to a care level according to the German nursing insurance system^(^ ^20^ ^)^ and had no terminal illness.

The fourth study was conducted in two municipal nursing homes (NH, *n* 200) in Nuremberg in 2007^(^ ^21^ ^)^. Participants with terminal illness, with acute diseases associated with hospital stays and with tube-feeding were excluded.

For the current secondary analysis, only study participants with a complete set of anthropometric data (body weight, height and self-reported weight loss) were included. Participants with >20 % missing values within a defined set of risk factors of malnutrition (see below) were excluded. The final samples included 1073 CD older adults, 180 GDH patients, 335 HC receivers and 197 NH residents.

### Definition of malnutrition

The analyses focused on protein–energy malnutrition which was defined as BMI < 20 kg/m^2^ and/or reported weight loss of >3 kg in the previous 3 to 6 months, following the German guidelines for clinical nutrition in geriatrics^(^ ^22^ ^)^.

Weight was measured with calibrated standing scales in CD older adults (SECA 635, Hamburg, Germany), GDH patients and HC receivers (Beurer PS 07, Ulm, Germany). In NH residents, weight was assessed with a calibrated weigh chair scale (Arjo CFA 2000, Mainz-Kastel, Germany). Height was measured by a conventional or an ultrasound stadiometer (Soehnle Professional, Backnang, Germany). In participants unable to stand upright (HC, NH), knee height was measured by a sliding calliper and height was calculated by the formulas of Chumlea *et al*.^(^ ^23^ ^)^. If measurements were not feasible data were taken from medical records. BMI was calculated as [weight (kg)]/[height (m)]^2^. Self-reported weight loss was assessed within the previous 6 months (CD: Seniors in the Community: Risk Evaluation for Eating and Nutrition, version II (SCREEN II)^(^ ^24^ ^)^; GDH: study-specific question) or the previous 3 months (HC, NH: Mini Nutritional Assessment (MNA)^(^ ^25^ ^)^).

### Participants’ characteristics and potential risk factors

Information on participants’ characteristics and potential risk factors of malnutrition were obtained by standardized questionnaires in all studies. In cases of severe cognitive impairment or inability to communicate, questions were addressed to the participants’ primary caregiver (HC) or the nursing staff (NH). Twenty-three potential risk factors were assessed identically or similarly in all four studies. When necessary, variable coding was harmonized after discussion and consent within the working group (E.K., M.G.C., D.V., C.M.). An overview on the harmonization of variable coding can be found in the online supplementary material, Supplemental Table S1.

Demographics are represented by age, gender and living situation. Living situation (alone *v*. with others) was considered in CD older adults, GDH patients and HC receivers, but not in NH residents.

Health status was characterized by polypharmacy (>3 prescribed drugs), multimorbidity (≥2 diagnosed chronic diseases) and the diagnosis of eight specific chronic diseases, namely diabetes mellitus, heart diseases, stroke, cancer, respiratory diseases, gastrointestinal diseases, renal diseases and arthropathy.

Mental function was evaluated by two variables. Cognitive impairment was categorized as Mini Mental State Examination (MMSE)^(^ ^26^ ^)^ score of <24 points in the GDH, HC and NH studies and as Telephone Interview for Cognitive Status–modified (TICS-m)^(^ ^27^ ^)^ score of ≤31 points in the CD study. Emotional status was assessed by the Geriatric Depression Scale (GDS)^(^ ^28^ ^)^ in all four studies. Scores >5 points indicated depressive symptoms.

Physical function was defined by having mobility limitations and eating difficulties using the respective items of the Barthel Index for activities of daily living^(^ ^29^ ^)^ in the GDH, HC and NH studies and the German version of the Health Assessment Questionnaire (HAQ)^(^ ^30^ ^)^ in the CD study. Items of both instruments were dichotomized (see Supplemental Table S1).

Dietary intake-related problems were defined as having poor appetite, nausea, chewing problems and swallowing problems. Nausea and chewing problems were assessed by study-specific questions. For poor appetite and swallowing problems, items from SCREEN II^(^ ^24^ ^)^ (CD) or study-specific questions (GDH, HC, NH) were used. All variables were dichotomized (see Supplemental Table S1).

Dietary behaviour was represented by two variables on low fruit and vegetable intake (<2 servings/d) and low fluid intake (<3 glasses/d). To build these variables the respective items of the MNA^(^ ^25^ ^)^ were used for the GDH, HC and NH studies and the items of SCREEN II^(^ ^24^ ^)^ for the CD study. All variables were dichotomized (see Supplemental Table S1).

### Data analysis

Missing data of eleven potential risk factors of malnutrition ranging from 0·1 to 6·3 % were imputed for each study separately using the interactive Markov chain Monte Carlo method. Twenty imputation models were created and in each model all variables were considered as both dependent and predictive variable. Regarding depressive symptoms, missing values were modelled as a separate category in HC and NH participants due to the high number of missing values because of inability to communicate or severe cognitive deficits (test not feasible). Results are presented for each setting (CD, GDH, HC and NH). Participants’ characteristics are given as mean and sd or as median and interquartile range for continuous variables and as relative frequencies for binary variables. Differences between studies were tested by the Kruskal–Wallis test followed by pairwise comparisons with Bonferroni correction for continuous variables and the *χ* ^2^ test followed by the *z* test with Bonferroni correction for nominal variables. Results were considered statistically significant when *P* values were <0·05 (two-sided). Univariate logistic regression analyses were performed for all twenty-three variables to identify factors associated with malnutrition in each of the four studies. Factors showing *P* < 0·10 were considered for the multivariable logistic regression models, which were built based on a command for stepwise forward inclusion of variables (likelihood ratio). Multicollinearity between variables significantly associated with malnutrition in the univariate approach was checked by calculating the variance inflation factor. As the variance inflation factor values were all about 1, multicollinearity was not assumed. Goodness-of-fit was evaluated by the Hosmer–Lemeshow test and explained variance was based on Nagelkerke’s *R* ^2^. In addition, the association between the number of potential individual risk factors and malnutrition was investigated by logistic regression analyses for each setting. The number of individual risk factors was calculated based on the twenty potential risk factors from the domains of health status, mental function, physical function, dietary intake-related problems and dietary behaviour. Demographics were not considered as living alone was not assessed in all four studies and age and gender were used as adjustment variables in the regression models. Statistical analysis was performed with the statistical software package IBM SPSS Statistics version 24.

## Results

### Prevalence of malnutrition

**Fig. 1:**
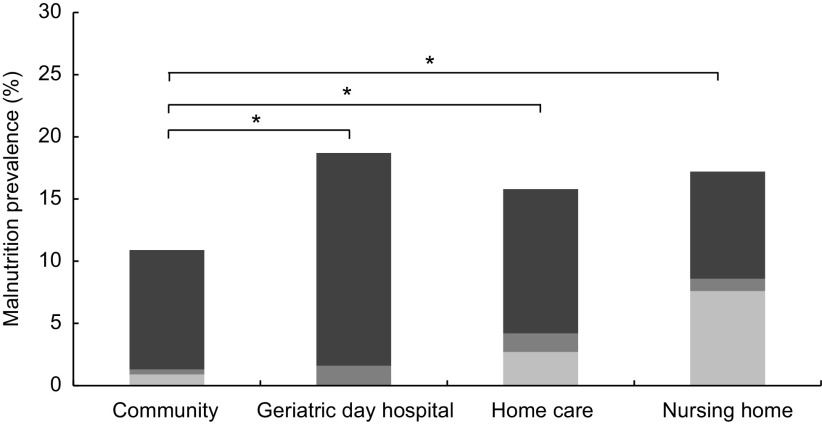
Prevalence of malnutrition (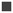, weight loss >3 kg in the previous 3–6 months; 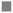, BMI < 20 kg/m^2^ plus weight loss >3 kg in the previous 3–6 months; 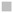, BMI < 20 kg/m^2^) in the samples of community-dwelling older adults (*n* 1073), patients of a geriatric day hospital (*n* 180), receivers of home care (*n* 335) and nursing home residents (*n* 197); secondary data analysis of studies conducted among adults aged ≥65 years, Germany, in 2009, 2012, 2010 and 2007, respectively. *P < 0·05 (*χ* ^2^ test)

The prevalence of malnutrition was 11·0 % in CD older adults, 18·9 % in GDH patients, 15·8 % in older adults receiving HC and 17·2 % in NH residents (Fig. 1). Prevalence was significantly lower in the CD sample compared with the three other settings. Across all settings participants were predominantly identified as malnourished due to weight loss; only in the NH sample was a distinct part of the participants (7·6 %) categorized as malnourished solely due to BMI < 20 kg/m^2^.

### Participants’ characteristics and potential risk factors of malnutrition

In Table 1 participants’ characteristics and potential risk factors of malnutrition are presented for each setting.

**Table 1:** Participants’ characteristics and potential risk factors of malnutrition for community-dwelling older adults, patients of a geriatric day hospital, receivers of home care and nursing home residents; secondary data analysis of studies conducted among adults aged ≥65 years, Germany, in 2009, 2012, 2010 and 2007, respectively

	Community (*n* 1073)		Geriatric day hospital (*n* 180)		Home care (*n* 335)		Nursing home (*n* 197)		*P*
Demographics									
Age (years)*	76·0^a^	6·6	79·3^b^	6·2	80·9^b^	7·7	85·5^c^	7·9	0·001
Female gender (%)	50·2^a^		71·7^b^		63·6^b^		73·6^b^		0·001
Living alone (%)	33·5^a^		63·8^b^		39·7^a^		–		0·001
Health status									
Polypharmacy (%)	54·9^a^		87·2^b^		83·9^b^		82·7^b^		0·001
Multimorbidity (%)	50·6^a^		84·4^b^		85·7^b^		83·2^b^		0·001
Diabetes mellitus (%)	17·6^a^		40·6^b^		28·1^c^		35·0^b,c^		0·001
Heart diseases (%)	31·4^a^		45·0^b^		73·7^c^		76·6^c^		0·001
Stroke (%)	8·5^a^		12·8^a^		29·9^b^		28·9^b^		0·001
Cancer (%)	4·4^a^		7·2^a,b^		12·2^b^		7·1^a,b^		0·001
Respiratory diseases (%)	10·7^a^		16·7^a,b^		26·6^b^		17·3^a,b^		0·001
Gastrointestinal diseases (%)	8·9^a^		6·7^a^		16·7^b^		15·7^b^		0·001
Renal diseases (%)	4·7^a^		28·9^b^		11·9^c^		18·8^b,c^		0·001
Arthropathy (%)	17·9^a^		42·2^b^		54·9^c^		31·0^b^		0·001
Mental function									
Cognitive impairment (%)	22·7^a^		11·7^b^		40·7^c^		65·7^d^		0·001
Depressive symptoms (%)	10·8^a^		30·2^b^		37·0^b^		35·2^b^		0·001
Depressive symptoms not assessed (%)†	0^a^		0^a^		12·9^b^		23·4^c^		
Physical function									
Mobility limitations (%)	17·1^a^		42·8^b^		56·4^c^		66·0^c^		0·001
Difficulties with eating (%)	9·0^a^		9·8^a^		43·6^b^		59·9^c^		0·001
Dietary intake-related problems									
Nausea (%)	17·8^a^		15·0^a^		27·7^b^		11·2^a^		0·001
Chewing problems (%)	19·5^a^		10·0^b^		16·2^a,b^		14·8^a,b^		0·002
Swallowing problems (%)	23·9^a^		13·9^b^		28·1^a^		12·3^b^		0·001
Poor appetite (%)	9·5^a^		25·6^b^		37·3^c^		35·4^b,c^		0·001
Dietary behaviour									
Low fruit/vegetable intake (%)	13·8^a^		35·6^b^		16·1^a^		33·9^b^		0·001
Low fluid intake (%)	2·2^a^		2·2^a^		1·5^a^		3·6^a^		0·293
Total number of risk factors‡	3^a^	2–5	6^b^	4–7	7^c^	6–9	7^c^	5–9	0·001

Comparisons between settings used the Kruskal–Wallis test followed by pairwise comparisons with Bonferroni correction for continuous variables and the *χ*^2^ test followed by the *z* test with Bonferroni correction for nominal variables.

^a,b,c,d^Mean or percentage values within a row with unlike superscript letters were significantly different in *post hoc* tests.

* Data presented as mean and sd.

† Depressive symptoms were assessed with the Geriatric Depression Scale. The application was not feasible in forty-two home-care receivers and thirty-eight nursing home residents due inability to communicate or severe cognitive deficits.

‡ Maximum number of potential individual risk factors (*n* 20) calculated based on the domains of health status, mental function, physical function, dietary intake-related problems and dietary behaviour, not including age, gender, living alone, presented as median and interquartile range.

#### Demographics

Mean age of participants was 76·0 years in CD older adults, 79·3 years in GDH patients, 80·9 years in HC receivers and 85·5 years in NH residents. The proportion of females was smaller in the CD sample (50·2 %) than in the other samples. Significantly more GDH patients lived alone compared with CD older adults and HC receivers.

#### Health status

Polypharmacy and multimorbidity were identified in about 50 % of the CD sample and in more than 80 % of the GDH, HC and NH samples. Accordingly, the prevalence of most single chronic diseases was also lower in the CD sample than in the other samples.

#### Mental function

The prevalence of cognitive impairment was lowest in GDH patients (10 %) and was about four and six times higher in HC and NH participants, respectively. Depressive symptoms were observed in about 11 % of CD older adults and did not differ between the other three studies with proportions above 30 %. Due to severe cognitive impairment, administration of the GDS was not feasible in 13 and 23 % of HC receivers and NH residents, respectively.

#### Physical function

The prevalence of mobility limitations was lowest in CD older adults (17 %) and highest in NH residents with two-thirds of the participants affected. Difficulties with eating were a minor problem in the CD and GDH samples (<10 %), while in the samples depending on care 44 % (HC) and 60 % (NH) were affected.

#### Dietary intake-related problems

Nausea was more prevalent in the HC sample than in all other samples. The prevalence of chewing problems varied slightly between the four samples and was lowest in GDH patients. Swallowing problems were reported more often in CD older adults and HC receivers than in GDH patients and NH residents. The prevalence of reduced appetite was distinctly lower in CD older adults compared with the other samples.

#### Dietary behaviour

Low fruit intake was more often present in GDH patients and NH residents than in CD older adults and HC receivers. Low fluid intake was only rarely reported in all four studies.

### Factors related to malnutrition

Table 2 shows the results of the univariate logistic regression analyses of the twenty-three potential risk factors of malnutrition for the four settings. Table 3 presents factors which were associated with malnutrition in the stepwise logistic regression models. In the CD sample, poor appetite, difficulties with eating, respiratory diseases and gastrointestinal diseases were identified as factors related to malnutrition. In GDH patients, poor appetite and respiratory diseases increased the likelihood of being malnourished. In the HC setting, lower age, poor appetite and nausea were associated with malnutrition. In the NH setting, higher age and mobility limitations remained as factors related to malnutrition.

**Table 2:** Results of the univariate logistic regression analyses to identify factors associated with malnutrition in community-dwelling older adults, patients of a geriatric day hospital, receivers of home care and nursing home residents; secondary data analysis of studies conducted among adults aged ≥65 years, Germany, in 2009, 2012, 2010 and 2007, respectively

	Community (*n* 1073)		Geriatric day hospital (*n* 180)		Home care (*n* 335)		Nursing home (*n* 197)	
	OR	95 % CI	OR	95 % CI	OR	95 % CI	OR	95 % CI
Demographics								
Age	**1**·**03**	**0**·**99, 1**·**06**	**0**·**94**	**0**·**89, 1**·**00**	**0**·**95**	**0**·**92, 0**·**99**	**1**·**05**	**1**·**00, 1**·**11**
Female gender	1·09	0·74, 1·60	0·57	0·26, 1·25	0·71	0·39, 1·28	1·84	0·71, 4·72
Living alone	**1**·**44**	**0**·**98, 2**·**12**	0·89	0·41, 1·93	**0**·**49**	**0**·**26, 0**·**95**	–	–
Health status								
Polypharmacy	**1**·**47**	**0**·**99, 2**·**19**	1·12	0·36, 3·54	0·79	0·37, 1·70	1·26	0·45, 3·52
Multimorbidity	**1**·**46**	**0**·**99, 2**·**16**	3·46	0·78, 15·38	1·37	0·55, 3·41	0·93	0·35, 2·45
Diabetes mellitus	0·87	0·51, 1·47	1·03	0·48, 2·20	0·91	0·47, 1·76	0·73	0·33, 1·64
Heart diseases	1·31	0·88, 1·95	0·83	0·39, 1·76	1·26	0·63, 2·53	1·21	0·49, 3·00
Stroke	1·55	0·85, 2·84	0·89	0·28, 2·81	1·25	0·67, 2·34	0·86	0·38, 1·98
Cancer	1·81	0·83, 3·97	**2**·**97**	**0**·**91, 9**·**75**	1·60	0·71, 3·58	2·04	0·60, 6·94
Respiratory diseases	**2**·**30**	**1**·**39, 3**·**80**	**2**·**63**	**1**·**09, 6**·**30**	**1**·**87**	**1**·**01, 3**·**47**	1·62	0·66, 3·97
Gastrointestinal diseases	**2**·**57**	**1**·**52, 4**·**36**	1·47	0·38, 5·76	**1**·**81**	**0**·**89, 3**·**66**	1·50	0·59, 3·83
Renal diseases	1·60	0·73, 3·49	0·86	0·37, 2·00	**1**·**95**	**0**·**89, 4**·**28**	1·74	0·73, 4·12
Arthropathy	**1**·**52**	**0**·**96, 2**·**40**	0·57	0·27, 1·31	0·76	0·42, 1·36	1·08	0·49, 2·39
Mental function								
Cognitive impairment	**1**·**51**	**0**·**96, 2**·**36**	1·01	0·32, 3·23	1·55	0·86, 2·81	1·40	0·59, 3·35
Depressive symptoms	**1**·**75**	**1**·**01, 3**·**02**	1·21	0·54, 2·71	**3**·**23**	**1**·**66, 6**·**28**	1·40	0·55, 3·58
Depressive symptoms not assessed*	**–**	**–**	**–**	**–**	1·44	0·50, 4·13	1·93	0·74, 5·03
Physical function								
Mobility limitations	**2**·**43**	**1**·**58, 3**·**73**	1·06	0·50, 2·26	1·21	0·67, 2·10	**3**·**56**	**1**·**31, 9**·**68**
Difficulties with eating	**3**·**56**	**2**·**16, 5**·**86**	**2**·**97**	**0**·**99, 8**·**84**	1·30	0·72, 2·34	1·76	0·79, 3·92
Dietary intake-related problems								
Nausea	**1**·**88**	**1**·**19, 2**·**96**	1·28	0·47, 3·45	**2**·**79**	**1**·**51, 5**·**10**	0·73	0·20, 2·63
Chewing problems	**1**·**90**	**1**·**18, 3**·**05**	0·85	0·23, 3·10	1·45	0·69, 3·03	1·89	0·73, 4·93
Swallowing problems	1·17	0·76, 1·81	1·09	0·38, 3·14	**2**·**07**	**1**·**13, 3**·**80**	2·01	0·74, 5·52
Poor appetite	**3**·**30**	**2**·**02, 5**·**43**	**7**·**82**	**3**·**46, 17**·**66**	**4**·**15**	**2**·**23, 7**·**72**	**2**·**55**	**1**·**19, 5**·**48**
Dietary behaviour								
Low fruit/vegetable intake	1·34	0·80, 2·24	1·56	0·73, 3·33	**2**·**46**	**1**·**24, 4**·**89**	1·05	0·47, 2·36
Low fluid intake	0·45	0·16, 1·25	0·69	0·07, 6·87	0·75	0·08, 6·83	–	–

Significant results are shown in bold.

* Depressive symptoms were assessed with the Geriatric Depression Scale. The application was not feasible in forty-two home-care receivers and thirty-eight nursing home residents due inability to communicate or severe cognitive deficits.

**Table 3:** Results of the stepwise logistic regression analyses to identify risk profiles associated with malnutrition in community-dwelling older adults, patients of a geriatric day hospital, receivers of home care and nursing home residents; secondary data analysis of studies conducted among adults aged ≥65 years, Germany, in 2009, 2012, 2010 and 2007, respectively

	OR	95 % CI	Model fit
Community (*n* 1073)*			
Poor appetite	2·42	1·43, 4·10	*χ* ^2^ = 47·72 −2 log likelihood = 691·6 Hosmer–Lemeshow test *P* = 0·800 *R* ^2^(Nagelkerke) = 0·087
Difficulties with eating	2·78	1·64, 4·70	
Gastrointestinal diseases	2·36	1·37, 4·08	
Respiratory diseases	1·88	1·11, 3·12	
Geriatric day hospital (*n* 180)†			
Poor appetite	8·01	3·48, 18·44	*χ* ^2^ = 29·8 −2 log likelihood = 144·7 Hosmer–Lemenshow test *P* = 0·997 *R* ^2^(Nagelkerke) = 0·246
Respiratory diseases	2·81	1·05, 7·48	
Home care (*n* 335)‡			
Age	0·95	0·91, 0·99	*χ* ^2^ = 36·23 −2 log likelihood = 256·3 Hosmer–Lemeshow test *P* = 0·239 *R* ^2^(Nagelkerke) = 0·176
Poor appetite	3·99	2·10, 7·58	
Nausea	2·39	1·27, 7·57	
Nursing home (*n* 197)§			
Age	1·06	1·00, 1·17	*χ* ^2^ = 12·2 −2 log likelihood = 169·1 Hosmer–Lemeshow test *P* = 0·887 *R* ^2^(Nagelkerke) = 0·099
Mobility limitations	3·70	1·35, 10·16	

* Not included in model by stepwise procedure: age, living alone, polypharmacy, multimorbidity, arthropathy, cognitive impairment, depressive symptoms, mobility limitations, nausea, chewing problems.

† Not included in model by stepwise procedure: age, cancer, difficulties with eating.

‡ Not included in model by stepwise procedure: living alone, respiratory diseases, gastrointestinal diseases, renal diseases, depressive symptoms, swallowing problems, low fruit/vegetable intake.

§ Not included in model by stepwise procedure: appetite.

### Number of risk factors

**Fig. 2:**
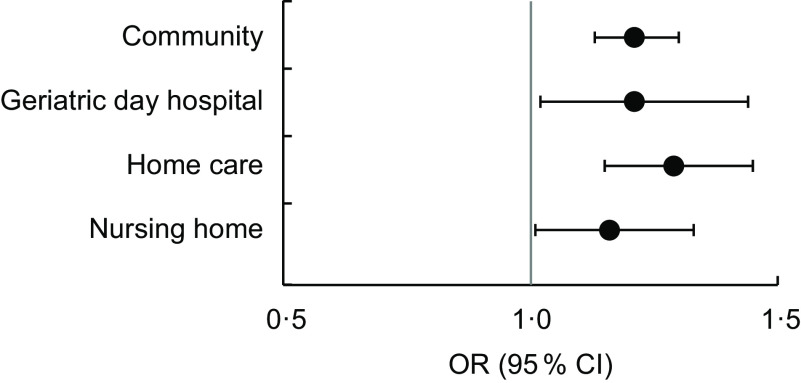
Association between the number of potential risk factors (*n* 20) and malnutrition in the samples of community-dwelling older adults (*n* 1073), patients of a geriatric day hospital (*n* 180), receivers of home care (*n* 335) and nursing home residents (*n* 197); secondary data analysis of studies conducted among adults aged ≥65 years, Germany, in 2009, 2012, 2010 and 2007, respectively. OR with their 95 % CI represented by vertical bars, adjusted for age and gender

The number of individual risk factors (median (interquartile range)) varied between settings and was higher in the HC (7 (6–9)) and NH studies (7 (5–9)) than in the GDH study (6 (4–7)) and the CD study (3 (2–5)), where it was lowest (Table 1). In all regression models the odds (OR (95 % CI)) of being malnourished increased with increasing number of potential individual risk factors (Fig. 2): CD, 1·21 (1·13, 1·30); GDH, 1·21 (1·02, 1·44); HC, 1·29 (1·15, 1·45); NH, 1·16 (1·01, 1·33).

## Discussion

The present study highlights risk profiles of malnutrition in older people from different settings and shows that the rate of older people being malnourished is increasing with the number of individual risk factors.

The four groups of participants included reflect the heterogeneity of the older population with respect to health and functional states (Table 1). Even though CD older adults were the youngest and less functionally impaired group, about 50 % of the sample was multimorbid and took more than three drugs, and 20–25 % were affected by cognitive impairment and swallowing problems. GDH patients, being vulnerable of becoming dependent on care but still living independently in the community, were distinctly more often than CD older adults affected by multimorbidity, polypharmacy, depressive symptoms and limitations in physical function. As both HC receivers and NH residents belong to the population dependent on care, especially physical and mental functional status were impaired compared with the other samples. In the HC setting the number of participants with nausea (28 %) was noticeable, which was higher compared with another study in the same setting reporting 17 % suffering from nausea^(^ ^31^ ^)^. The age of NH residents was on average 10 years older than that of CD older adults. This difference is also described by other studies conducted in the respective settings^(^ ^5^ ^,^ ^32^ ^)^. Differences between the settings are also stressed by the varying number of potential individual risk factors of malnutrition. While the median number was only three in the CD sample, it was more than doubled in the HC and the NH samples.

The prevalence of malnutrition varied likewise between the settings and was significantly lower in CD older adults than in the other three settings. However, as the majority of older people live independently in the community, this is a very relevant group regarding the prevention and treatment of malnutrition. Cereda *et al*. used the MNA to define malnutrition in their meta-analysis^(^ ^5^ ^)^. Using BMI and weight loss we identified higher prevalence for CD older adults, GDH patients and HC receivers, while the prevalence for NH residents was found to be similar. An explanation for these different malnutrition rates might be the fact that the MNA considers, in addition to anthropometric measures, health and functional aspects^(^ ^33^ ^)^. A Dutch study, using also low BMI and weight loss as parts of the definition of malnutrition, but with deviating cut-offs from our study, reported a slightly higher prevalence in receivers of HC (22 %) and NH residents (19 %) compared with our data (Fig. 1)^(^ ^15^ ^)^. In another German study with patients of a GDH^(^ ^34^ ^)^ the prevalence of a low BMI was 2 % and similar to our results. The distinctly higher proportion of participants with weight loss in the last 3 months (42 %) within the former study^(^ ^34^ ^)^ can be explained by not specifying a cut-off for a lower limit of weight loss. Regarding the criteria used to define malnutrition in our samples weight loss played the major role, while a low BMI became relevant only with increasing need of care, especially in the NH setting. However, compared with reviews reporting prevalence rates for BMI < 20 kg/m^2^ of between 9 and 40 % in NH residents^(^ ^8^ ^,^ ^35^ ^)^, the number found in our study (8 %) was low. The differences might be due to sampling characteristics.

The stepwise logistic regression analyses identified the most relevant factors associated with malnutrition in each setting. The setting-specific risk profiles represent only small sets of variables (two to four) and do not cover all domains of investigated risk factors. Generally, the factors subsumed under the domains mental function and dietary behaviour seemed to be of minor importance for the occurrence of malnutrition in all four settings. The CD setting presented the most diverse set of risk factors covering all remaining domains but demographics. In the GDH setting the two identified risk factors from the health and the dietary intake-related problems domains, namely respiratory diseases and poor appetite, overlap with the risk profile of the CD older adults. In the HC setting dietary intake-related problems were of primary importance and in the NH setting, besides age, physical functional aspects played the major role.

From the domain of dietary intake-related problems, poor appetite was identified as a major factor related to malnutrition in our study with especially high OR in the GDH setting. This result is in accordance with the systematic review of van der Pols-Vijlbrief *et al*. reporting strong evidence for an association between poor appetite and malnutrition in CD older adults^(^ ^12^ ^)^. Also studies within the NH setting reported an association between poor appetite and malnutrition^(^ ^36^ ^,^ ^37^ ^)^, while a study in HC receivers did not^(^ ^31^ ^)^. Poor appetite seems to be an important trigger for reduced dietary intake in older people and is a consequence of age-related physiological changes, diseases, depression, medication use, lower physical activity and social isolation^(^ ^38^ ^,^ ^39^ ^)^. Nausea was the second dietary intake-related problem found to be associated with malnutrition; however, in the multivariate analyses the association remained significant only in HC receivers. The relevance of nausea in the HC setting is supported by its prevalence and by a Dutch study in older adults receiving HC also observing an association with (risk of) malnutrition^(^ ^31^ ^)^.

From the health domain, respiratory diseases were associated with malnutrition in CD older adults as well as in GDH patients and gastrointestinal diseases in CD older adults. Both diseases are linked to inflammation, increased energy demands and wasting conditions in severe disease stages, which possibly explains the link to malnutrition^(^ ^40^ ^–^ ^42^ ^)^. There are few other studies using similar statistical approaches showing an association of gastrointestinal diseases with malnutrition in different community settings^(^ ^15^ ^,^ ^31^ ^)^. However, in a systematic review no conclusions for an association of both respiratory and gastrointestinal diseases with malnutrition were drawn due to limited studies^(^ ^12^ ^)^. In all four settings polypharmacy and multimorbidity were not found to be associated with malnutrition in the multivariate statistical models. This is in line with the results of several other studies from different settings^(^ ^43^ ^–^ ^45^ ^)^. The lack of association might be due to little variation in these variables as up to 87 % of the participants were affected. Only in the CD setting, in which the prevalence was lower (50 %), was a tendency towards an association visible in the univariate approach. The health factors diabetes mellitus, heart diseases and stroke were consistently not associated with malnutrition in all statistical models indicating a low relevance of these factors in the respective settings. The results are in line with those of the several other investigations showing no or inverse associations between these diseases and malnutrition^(^ ^12^ ^,^ ^15^ ^)^. Stroke as a potential risk factor of malnutrition might be more relevant in acute and post-acute settings; and furthermore, stroke patients dependent on tube-feeding, and therefore potentially at higher risk of malnutrition, might be under-represented in investigations.

In the functional domains only physical but not mental functional factors were related to malnutrition in our study. Although difficulties with eating were distinctly more prevalent in the samples dependent on care (HC, NH), they were identified as a risk factor of malnutrition only in CD older adults. A reason could be that in the HC setting caregivers are aware of the problems and provide adequate help, while CD older adults have presumably no or less support from caregivers and cannot cope with the problems. In several other studies using different definitions of malnutrition, eating dependency was also reported as a risk factor in older adults living in the community, sheltered housing and nursing homes^(^ ^16^ ^,^ ^36^ ^,^ ^46^ ^)^. The association between mobility limitations and malnutrition seems to be moderated by the factor setting in our analyses. Mobility limitations were identified as a factor related to malnutrition only in the NH study, while in the CD setting as well as in the combined sample the univariate association was attenuated in the multivariate analyses. It is surprising that mobility limitations seem to be a less relevant risk factor of malnutrition in the GDH and HC samples, as it could be expected that mobility limitations impede shopping and cooking and therefore the supply of foods. This could be a sign for an adequate support by family members and caregivers regarding shopping and meal preparation in these samples. In other studies mobility limitations were found to be associated with different surrogates of malnutrition in CD, HC and NH samples^(^ ^14^ ^,^ ^31^ ^,^ ^47^ ^,^ ^48^ ^)^. The conflicting results might be partly due to different assessment methods of mobility and a more detailed assessment of the mobility status might be needed to show an association with malnutrition. Regarding mental function, cognitive impairment and depressive symptoms were univariately related to malnutrition in the CD and the HC samples (only depressive symptoms) but the effects were attenuated in the multivariable models. Previous results from other studies are inconsistent. In a recent multi-cohort meta-analysis in CD older adults both aspects were not associated with incident malnutrition^(^ ^48^ ^)^. In the review of van der Pols-Vijlbrief *et al*.^(^ ^12^ ^)^, also focusing on CD adults, moderate evidence for no association between depression and malnutrition and inconclusive results for cognitive status were reported, while the review of Tamura *et al*.^(^ ^14^ ^)^ in NH residents found both depression and cognitive limitations to be associated with malnutrition.

From the demographic domain, only age was identified as a factor associated with malnutrition in HC receivers and NH residents but not in CD older adults and GDH patients. The direction of the association differed between the two settings. In HC receivers higher age paradoxically reduces the likelihood of malnutrition, which is consistent with a Dutch study in older HC receivers^(^ ^47^ ^)^. Also in line with our findings Tamura *et al*.^(^ ^14^ ^)^ reported higher age to be a risk factor of malnutrition in NH residents, and van der Pols-Vijlbrief *et al*.^(^ ^12^ ^)^ found moderate evidence for no association between age and malnutrition in CD older adults. An explanation for these discrepancies could be that usually with increasing age also chronic diseases and functional impairments occur more often^(^ ^49^ ^,^ ^50^ ^)^, which might lead to nursing home admission. Consequently, those ‘older olds’ in need of care, who remain home-dwelling, might represent a specific less morbid group with lower risk of malnutrition. Moreover, the HC sample might be influenced by ‘younger olds’ being dependent on care because of lifelong or accident-related disabilities and not due to age-associated functional decline and chronic diseases.

The factors of the domain dietary behaviour were not found to be related to malnutrition in all four settings. In a recently published study in CD older adults, diet quality according to the Healthy Eating Index was not associated with incident malnutrition^(^ ^51^ ^)^. However, other aspects of dietary behaviour, like protein intake, which could not be addressed in our study, have been shown to be related to malnutrition^(^ ^51^ ^)^.

Our results indicate that within all four settings the likelihood of being malnourished increased with the number of potential individual risk factors. The simultaneous occurrence of potential risk factors from the same or from different domains may aggravate the gap between (increased) energy and protein requirements and (reduced) intake, which is causal for the development of malnutrition.

Covering a broad age, health and functional spectrum, using a large set of potential risk factors, applying uniform definitions of malnutrition and risk factors to the four studies in different settings, and using the same statistical approach are strengths of the current secondary data analysis. However, some limitations need to be addressed. First, the analysis was based on cross-sectional data only, not allowing the assumption of any causal relationships, and the sample sizes of some studies were small resulting in a lower power of the analyses which could be responsible for some of the differences found between studies. Second, the analysis is limited to the exploration of potentially individual risk factors that were assessed in all four studies similarly. Therefore, some further potentially important factors addressing the individual level, like income or loneliness, as well as interpersonal or environmental levels might be missing. Third, when harmonizing the data of the four studies, a reduction of scale levels was necessary for some variables to achieve comparability, which leads to a loss of information. In addition, the type of assessment (e.g. self- *v*. proxy report) was partly different between studies. Fourth, to define weight loss we used the respective question from the applied malnutrition screening tools. As these questions do not specifically ask for unintentional weight loss, we cannot completely rule out that some participants lost weight intentionally. Moreover, it needs to be considered that we used weight loss in kilograms to define malnutrition, while guidelines suggest using percentage weight loss^(^ ^52^ ^)^. Regarding the generalizability of our results it needs to be considered that populations referring to the four investigated settings might vary between countries because of different health-care systems.

## Conclusion

The results of the present study stress differences in the prevalence of malnutrition and its potential risk factors between four geriatric settings and indicate a varying relevance of specific factors associated with malnutrition in the four settings. The relationship between the number of individual risk factors and malnutrition in all settings implies comprehensive assessment approaches for clinical practice, considering not only single risk factors but also their combination to identify persons at risk of malnutrition early. To better understand the complex aetiology of malnutrition in older adults, the prognostic value of the identified factors regarding the development of malnutrition should be the subject of future longitudinal studies in the different settings. In addition, research needs to be further aligned by establishing uniform diagnostic criteria of malnutrition and by defining a minimum data set covering all relevant domains of risk factors, which are assessed in a standardized way.

## Supplementary material

For supplementary material accompanying this paper visit https://doi.org/10.1017/S1368980019002271. click here to view supplementary material
