# Supplementary material for: Malnutrition and related risk factors in older adults from different health-care settings: an enable study
Source: Public Health Nutr. 2019 Aug 27;23(3):446–56. doi: 10.1017/S1368980019002271 (PMC7025158; doi:10.1017/S1368980019002271)
Supplement: Supplementary file 1 [file S1368980019002271sup.zip › S1368980019002271sup002.pdf]

Supplementary File 1: Overview on the harmonization of the 21 potential risk factors of malnutrition. Categorization used in the original studies (vs. indicates which categories were summarized during harmonization) as well as categorization used in this secondary data analyses is given

	Community (KORA-Age)	Geriatric Day Hospital	Home Care (ErnSiPP)	Nursing Home	Harmonized variable
**Demographics**					
Living alone	Do you live alone?<br>yes, no	Do you live alone?<br>yes, no	With whom does the home care receiver live together?<br>alone, with partner, with children, with partner and children, with other persons	-	Living alone, living with others
**Health Status**					
Polypharmacy	Number of prescribed drugs	Takes more than 3 prescription drugs per day<br>yes, no	Number of prescribed drugs	Number of prescribed drugs	> 3 drugs vs. ≤ 3 drugs
Multimorbidity	Number chronic diseases	Number chronic diseases	Number chronic diseases	Number chronic diseases	≥ 2 diseases vs. < 2 diseases
Diabetes Mellitus	Diabetes Mellitus<br>yes, no	Diabetes Mellitus<br>yes, no	Diabetes Mellitus<br>yes, no	Diabetes Mellitus<br>yes, no	Yes vs. no
Heart Diseases	Myocardial infarction<br>yes, no<br>Heart complaint, heart diseases<br>yes, no	Heart failure<br>yes, no<br>Other heart diseases<br>yes, no	Heart failure<br>yes, no<br>Other heart diseases<br>yes, no	Heart failure<br>yes, no<br>Other heart diseases<br>yes, no	Yes vs. no
Stroke	Stroke<br>yes, no	Stroke<br>yes, no	Stroke<br>yes, no	Stroke<br>yes, no	Yes vs. no
Cancer	Cancer (last 3 years)<br>yes, no	Cancer<br>yes, no	Cancer<br>yes, no	Cancer<br>yes, no	Yes vs. no

	Community (KORA-Age)	Geriatric Day Hospital	Home Care (ErnSiPP)	Nursing Home	Harmonized variable
Respiratory Diseases	Asthma, Emphysema, Chronic Obstructive Disease<br>yes, no	Respiratory Diseases<br>yes, no	Respiratory Diseases<br>yes, no	Respiratory Diseases<br>yes, no	Yes vs. no
GI diseases	GI diseases<br>yes, no	Gastritis, intestinal diseases<br>yes, no<br>Inflammatory Bowel disease<br>yes, no	Gastritis, intestinal diseases<br>yes, no<br>Inflammatory Bowel disease<br>yes, no	Gastritis, intestinal diseases<br>yes, no<br>Inflammatory Bowel disease<br>yes, no	Yes vs. no
Renal diseases	Renal diseases<br>yes, no	Renal diseases<br>yes, no	Renal diseases<br>yes, no	Renal diseases<br>yes, no	Yes vs. no
Arthropathy	Arthritis, Rheumatism, Arthrosis<br>yes, no	Arthropathy<br>yes, no	Arthropathy<br>yes, no	Arthropathy<br>yes, no	Yes vs. no
**Mental Function**					
Cognitive Impairment	TICS-m ≤ 31 points cognitive impairment	MMSE<br>< 24 points cognitive impairment	MMSE<br>< 24 points cognitive impairment	MMSE<br>< 24 points cognitive impairment	cognitive impairment vs. no cognitive impairment
Depressive Symptoms	Geriatric Depression Scale	Geriatric Depression Scale	Geriatric Depression Scale	Geriatric Depression Scale	> 5 points depressive symptoms vs. ≤ 5 points no depressive symptoms, not assessable
**Physical Function**					
Mobility Limitations	HAQ<br>Mobility limitations<br>Are you able to walk outside on flat ground?<br>unable to do, with much	Barthel Index<br>Mobility limitations<br>Can’t move at least 50 m, wheelchair bound, can move 50 m with support	Barthel Index<br>Mobility<br>Can’t move at least 50 m, wheelchair bound, can move 50 m with support	Barthel Index<br>Mobility<br>Can’t move at least 50 m, wheelchair bound, can move 50 m with support	yes vs. no

	Community (KORA-Age)	Geriatric Day Hospital	Home Care (ErnSiPP)	Nursing Home	Harmonized variable
	difficulty, with some difficulty **vs.** without any difficulty	**vs.** can move 50 m independently	**vs.** can move 50 m independently	**vs.** can move 50 m independently	
Difficulties with eating	HAQ<br>Mobility limitations<br>1. Are you able to cut meat with a knife?<br>2. Are you able to lift a full glass to the mouth?<br>unable to do, with much difficulty, with some difficulty **vs.** without any difficulty	Barthel Index<br>Eating difficulties<br>Completely dependent with eating, needs help with eating **vs.** eats independently	Barthel Index<br>Eating difficulties<br>Completely dependent with eating, needs help with eating **vs.** eats independently	Barthel Index<br>Eating difficulties<br>Completely dependent with eating, needs help with eating **vs.** eats independently	yes vs. no
**Dietary Intake Related Problems**					
Nausea	Nausea or stomach upset<br>yes, no	Do you suffer from nausea?<br>yes, no	Does the home care receiver suffer from nausea?<br>Often, occasionally, infrequently **vs.** never	Does the resident suffer from nausea?<br>yes, no	yes vs. no
Chewing problems	In the past month did you have difficulties with chewing of foods due to problems with teeth, oral problems or problems with dentures?<br>yes, no	Do you have difficulties with chewing?<br>yes, no	Does the home care receiver have difficulties with chewing?<br>Yes **vs.** only with hard foods, no	Does the resident have difficulties with chewing?<br>severe, moderate, slight **vs.** no	yes vs. no
Swallowing problems	Do you get cough, retch or pain during swallowing for foods or fluids?	Do you have swallowing difficulties?<br>yes, no	Does the home care receiver have swallowing difficulties?	Does the resident have swallowing difficulties?<br>severe, moderate, slight	yes vs. no

	Community (KORA-Age)	Geriatric Day Hospital	Home Care (ErnSiPP)	Nursing Home	Harmonized variable
	Often/almost always, occasionally, infrequently, never		yes, no	**vs.** no	
Poor appetite	How would you describe your appetite?<br>poor, less good **vs.** good, very good	Do you suffer from poor appetite?<br>yes, no	How would you describe the appetite of the home care receiver?<br>poor, moderate **vs.** good, very good	Does the resident suffer from poor appetite?<br>severely, moderately, slightly **vs.** no	yes vs. no
**Dietary Behavior**					
Low fruit/vegetable intake	How many serving of fruit, vegetables and salad do you usually per day? (SCREEN II)<br><2, 2, 3, 4, $\geq$ 5 servings	Consumes two or more servings of fruit or vegetables<br>per day? (MNA)<br>yes, no	Consumes two or more servings of fruit or vegetables<br>per day? (MNA)<br>yes, no	Consumes two or more servings of fruit or vegetables<br>per day? (MNA)<br>yes, no	<2 serving vs. $\geq$ 2 servings
Low fluid intake	How much do you drink usually per day? (SCREEN II)<br>$<$ 2 glasses, about 2 glasses, 3-4 glasses, 5-7 glasses, $\geq$ 8 glasses	How much fluid (water, juice, coffee, tea, milk ...) is consumed per day? (MNA)<br>$<$ 3 cups, 3-5 cups, $>$ 5 cups	How much fluid (water, juice, coffee, tea, milk ...) is consumed per day? (MNA)<br>$<$ 3 cups, 3-5 cups, $>$ 5 cups	How much fluid (water, juice, coffee, tea, milk ...) is consumed per day? (MNA)<br>$<$ 3 cups, 3-5 cups, $>$ 5 cups	$<$ 3 glasses vs. $\geq$ 3 glasses/cups

MMSE Mini Mental State Examination, TICS-m Telephone Interview for Cognitive Status-modified, HAQ Health Assessment Questionnaire, MNA Mini Nutritional Assessment
